# Electron Beam Irradiation Improves the Storage Quality of Passion Fruit by Modulating Membrane Lipid Composition and Enhancing ROS-Scavenging Potential

**DOI:** 10.3390/foods15122054

**Published:** 2026-06-06

**Authors:** Yazhen Chen, Yuzhao Lin, Minjun Lin, Xuanjing Jiang, Hongbin Chen

**Affiliations:** 1 College of Oceanology and Food Science, Quanzhou Normal University, Quanzhou 362000, China; cyz3368@163.com (Y.C.); linmj0426@163.com (M.L.); xuanlikemilk@126.com (X.J.); 2 Key Laboratory of Inshore Resources Biotechnology, Fujian Province University, Quanzhou 362000, China

**Keywords:** passion fruit, electron beam irradiation, storage quality, cell membrane, membrane lipids, ROS-scavenging capacity

## Abstract

This study investigated the impacts of electron beam irradiation (EBI) on the storage quality of passion fruit by regulating membrane lipid composition and reactive oxygen species (ROS)-scavenging capacity. Among the tested doses, 0.2 kGy EBI was the most effective in maintaining fruit quality, as indicated by lower relative electrical conductivity and weight loss, together with higher hue angle and commercially acceptable fruit rate during storage. Mechanistically, EBI reduced superoxide anion production and malondialdehyde accumulation, indicating alleviated oxidative damage and membrane lipid peroxidation. EBI also suppressed the activities of membrane lipid-degrading enzymes, thereby slowing the degradation of phospholipids and unsaturated fatty acids while reducing the accumulation of phosphatidic acid, diacylglycerol, and saturated fatty acids. In addition, EBI enhanced the activities of antioxidant enzymes and maintained higher levels of ascorbic acid, glutathione, and 1,1-diphenyl-2-picrylhydrazyl (DPPH) radical-scavenging ability. These results indicate that EBI improves the storage quality of passion fruit mainly by preserving membrane lipid integrity and strengthening ROS-scavenging capacity.

## 1. Introduction

Passion fruit (*Passiflora edulis* Sims), native to Brazil, acts as a perennial herbaceous vine belonging to the genus *Passiflora* in the family Passifloraceae [1]. Rapid expansion of passion fruit cultivation has been witnessed in recent years across southern China, covering Guangxi, Guangdong, Fujian, and Hainan [2,3]. In addition, passion fruit is a rich source of bioactive substances, such as phenolics, vitamins, amino acids, minerals, and carbohydrates [4,5,6], with its popularity and consumption stemming from the fruit’s unique aroma and nutritive value [7]. As a climacteric fruit, passion fruit exhibits active respiration, which accelerates the decay process, and triggers a series of quality deterioration processes, such as dehydration, wrinkling, microbial growth, or color darkening [1,7,8]. These postharvest problems not only cause a reduction in fruit quality and a short storage time, but also impair the fruit’s appearance and commercial value, ultimately resulting in economic losses [6,7]. Therefore, developing appropriate technologies to mitigate postharvest decay and improve storage quality in passion fruit is critical.

Studies have revealed that reactive oxygen species (ROS), such as superoxide anion (O_2_^−.^), are responsible for the quality decay and reduced storage quality of fruits [5,6]. Excessive accumulation of ROS not only oxidizes the unsaturated fatty acids (USFAs) on cell membranes of fruits [9], but also induces membrane lipid peroxidation, leading to structural impairment of cell membranes [10]. The integrity of the cell membrane is pivotal for sustaining cellular homeostasis [11]. Membrane lipids, including phospholipids such as phosphatidylcholine (PC) and phosphatidylinositol (PI), as well as fatty acids such as USFAs and saturated fatty acids (SFAs), are major structural components of cell membranes and are essential for normal cellular physiological metabolism [9,12]. Alterations in these lipids are a major cause of membrane damage and are regulated by membrane lipid metabolism-related enzymes (MLMREs), such as lipase (LPS), lipoxygenase (LOX), phospholipase D (PLD), PI-specific PLC (PI-PLC), and PC-specific PLC (PC-PLC) [11,12,13,14]. Specifically, PLD hydrolyzes PC into phosphatidic acid (PA) [15]. However, abnormal accumulation of PA can disrupt the structure of the membrane bilayer, thereby compromising the cell membrane integrity. The hydrolysis of PC is specifically mediated by PC-PLC, generating diacylglycerol (DAG) as a product, but PI undergoes enzymatic decomposition by PI-PLC, yielding DAG [13]. Furthermore, LPS further catalyzes the hydrolysis of DAG and PA into free fatty acids (FFAs), which consist of SFAs or USFAs [13]. Meanwhile, LOX catalyzes the oxidation of USFAs to generate SFAs [5,12]. Elevated activities of MLMREs can accelerate membrane lipid metabolism, promote the modification of membrane lipids, and consequently impair the structure of cell membranes. In a study by Yu et al. [11], the application of Tert-butylhydroquinone to longan fruit was shown to suppress membrane damages by inhibiting PLD, LOX, and LPS activities, and delaying the reduction in USFA contents. Moreover, spermidine inhibited the activities of PLD, LPS, LOX, phospholipase C (PLC), and phospholipase A (PLA), while reducing the breakdown of cellular membrane components, such as phospholipids and USFAs, thus preserving the quality of prune fruit [16]. Therefore, changes in membrane lipid compositions and integrity of cell membranes are closely associated with the quality of fruits.

To maintain ROS homeostasis, fruits have also evolved an effective ROS-scavenging system, comprising non-enzymatic as well as enzymatic components, alleviating the oxidative stress induced by ROS burst [6,17]. The enzymatic fractions of the ROS-scavenging system consist of several ROS-scavenging enzymes (RSEs), such as superoxide dismutase (SOD), catalase (CAT), peroxidase (POD), and ascorbate peroxidase (APX) [17,18,19]. As the first line of defense, SOD scavenges O_2_^−.^ and catalyzes its conversion into hydrogen peroxide (H_2_O_2_) [17,20]. Subsequently, excess H_2_O_2_ is eliminated through POD, APX, and CAT, which also play essential roles in eliminating oxygen radicals [17,21]. In addition to these enzymes, the non-enzymatic components, including ascorbic acid (AA) and glutathione (GSH), play crucial roles in ROS elimination, thereby maintaining redox balance in fruits [22,23]. AA may enhance APX activity to facilitate the elimination of H_2_O_2_, while GSH scavenges both H_2_O_2_ and other forms of ROS [5]. In a study conducted by Yang et al. [9], either methyl jasmonate or methyl salicylate was found to enhance the RSE activities (APX, SOD, CAT, etc.) in winter jujubes, thus preserving the storage quality and extending the storage time. Adenosine triphosphate enhanced the longan fruit quality by boosting the CAT, SOD, and APX activities, as well as GSH and AA contents, while decreasing the ROS levels [12]. Therefore, it is essential to enhance the ROS-scavenging potential of fruits, and to maintain low ROS levels for preserving their quality.

In the 1980s, the World Health Organization officially confirmed the safety of irradiated food, a move that further accelerated the global development of food irradiation and preservation industry. Irradiation preservation typically utilizes electron beam or γ-ray as radiation sources, with the latter emitted from ^137^Cs and ^60^Co. In recent years, amid rising cobalt source costs and the growing urgency of radioactive source decommissioning and disposal issues, the electron beam has gradually emerged as a viable alternative to other irradiation methods [24]. Unlike γ-ray irradiation, electron beam irradiation (EBI) generates no radioactive waste, and thus poses no concern regarding environmental pollution. Furthermore, EBI is regarded as the environmentally friendly, non-thermal food processing technology that may sterilize produce and extend its shelf life [24,25]. It offers several advantages: no requirement for load pretreatment; the absence of chemical residues; and a shorter sterilization exposure time and faster processing rate [25]. Some studies have shown that EBI could be used to reduce postharvest losses and enhance storage quality in mango fruit [26] and *Actinidia arguta* [19]. However, whether EBI improves the storage stability of passion fruit by regulating membrane lipid metabolism and ROS-scavenging capacity remains unclear. To the best of our knowledge, no previous study has systematically linked EBI-induced changes in membrane lipid composition, MLMRE activities, antioxidant defense responses, and storage quality in passion fruit. Therefore, the novelty of this study lies in identifying the optimal EBI dose for passion fruit preservation and elucidating its potential mechanism from the perspective of membrane lipid integrity and ROS homeostasis. In this study, we evaluated the effects of different EBI doses on key quality attributes, including relative electrical conductivity (REC), hue angle, commercially acceptable fruit rate, and weight loss rate. Subsequently, the optimal EBI treatment was selected to further investigate changes in ROS accumulation, lipid peroxidation, membrane lipid components, MLMREs, antioxidant enzymes, non-enzymatic antioxidants, and 1,1-diphenyl-2-picrylhydrazyl (DPPH) radical-scavenging ability. This study provides new insights into the postharvest application of EBI for maintaining passion fruit quality and clarifies the physiological basis underlying EBI-mediated storage stability.

## 2. Materials and Methods

### 2.1. Raw Material and Treatments

Passion fruit cv. Qinmi No. 9 was sourced from a commercial orchard located in Huian, Fujian Province, China. Upon completion of harvesting, the fruit was promptly transported to our laboratory for subsequent experiments. The passion fruits that displayed neither mechanical damage, pests, nor diseases, and that were of a relatively uniform shape, color, and maturity were selected. Then, the fruit was rinsed with distilled water and air-dried for later treatments.

A total of 30 fruits were utilized to determine fruit attributes at day 0. In addition, 900 fruits were divided into six groups based on irradiation doses and designated as follows: one control group (0 kGy EBI) and five EBI-treated groups with irradiation doses of 0.1, 0.2, 0.3, 0.4, and 0.5 kGy, corresponding to the absorbed doses. All the fruit samples were packed in polyethylene film bags with a thickness of 0.02 mm, after which they were subjected to irradiation treatments. The experimental EBI process was conducted at Fujian Quanzhou Quneng Technology Co., Ltd. For this process, the setup relied on a 10 MeV/20 kW high-energy electron accelerator (model: Nuctech IS1020), together with its supporting beam transport system and radiation shielding apparatus. After irradiation, the irradiated fruit was stored at 25 °C at a relative humidity of 85% for 15 d. Within the storage period, 30 samples per group were selected every 3 days to appraise the storage quality, with the aim of determining the optimal dose of EBI for enhancing the fruit quality. Furthermore, the underlying mechanism by which EBI at the optimal dose enhanced the storage quality of passion fruit, by regulating membrane lipid composition levels and ROS-scavenging capacity, was further investigated.

For each treatment and sampling time, fruits were randomly selected for physiological and biochemical analyses. All indicators in this study were measured in triplicate. Each biological replicate was prepared from independently sampled fruit tissues. For each biological replicate, pericarp tissues from five randomly selected fruits were pooled, immediately frozen in liquid nitrogen, ground into powder, and stored at −80 °C until analysis. Except for the assays of fruit storage quality, the powdered composite sample from each biological replicate was used independently for the determination of O_2_^−.^ production rate, malondialdehyde (MDA) content, enzyme activities, phospholipid contents, ROS-scavenging substance contents, and DPPH radical-scavenging ability using 1.0 g of pericarp tissue pooled from five fruits. For the determination of fatty acid amounts, 5.0 g of pericarp tissue pooled from five fruits was used.

### 2.2. Assay of Fruit Storage Quality

Thirty pericarp slices from five passion fruits of a uniform size of 0.2 cm^2^ were prepared as test samples. The REC was determined following Lin et al. [5] and Chen et al. [27], and the result was expressed as a percentage.

The hue angle value was recorded to reflect the fruit appearance, with reference to the procedure of Bai et al. [28] using a colorimeter (model: CHROMA METER CR-400).

Following Lin et al. [29], ten passion fruits were examined to determine the commercially acceptable fruit rate, which was defined as the proportion of fruit without disease or browning. Additionally, weight loss rate was computed by comparing the fruit weight at each assessment time with the initial weight recorded on day 0. Their results were expressed as percentage.

### 2.3. Assay of O_2_^−.^ Production Rate and MDA Amount

The O_2_^−.^ and MDA levels were determined in accordance with the protocol described by Lin et al. [30]. For the assay of O_2_^−.^ production rate, pericarp tissue (1.0 g, pooled from five fruits) was homogenized with 10 mL of 50 mmol L^−1^ phosphate buffered saline (PBS) buffer (pH 7.8) containing 1 mmol L^−1^ EDTA, followed by centrifugation at 12,000× *g* for 20 min at 4 °C. The reaction system was composed of 1 mL supernatant, 1 mL hydroxylamine hydrochloride, and 1 mL 50 mmol L^−1^ PBS. This mixture was then incubated at 25 °C for 60 min. Subsequently, 1 mL 7 mmol L^−1^ α-naphthyl amine and 1 mL 17 mmol L^−1^ p-aminobenzenesulfonic acid were added to the above mixture. After color development at 25 °C for 20 min, the absorbance was immediately measured at 530 nm using a microplate reader (Infinite M200 Pro, Tecan, Männedorf, Switzerland). For MDA determination, the pericarp tissue (1.0 g, pooled from five fruits) was homogenized with 5 mL of 10% trichloroacetic acid (TCA) and centrifuged under the same conditions. The reaction system was prepared by mixing 4 mL extract with an equal volume of 0.67% thiobarbituric acid, followed by heating the mixture for 20 min. After cooling, absorbance was recorded at 450, 532, and 600 nm. Their results were calculated based on the extraction volume and sample fresh weight and presented in μmol g^−1^ min^−1^ and nmol g^−1^, separately.

### 2.4. Assay of Related Enzyme Activities

The enzyme extracts of PLD, LPS, and LOX were prepared, and their respective activities were assayed, in accordance with the protocols established via Kuang et al. [10] and Li et al. [31]. In the PLD activity assay, its reaction mixture contained 3 mL enzyme extract and 3 mL lecithin as the substrate. For the assay of LPS activity, its reaction system included 1 mL enzyme extract as well as α-naphthyl acetate as the substrate, with 0.15% fast blue B salt added afterward as a chromogenic agent. For the determination of LOX activity, its reaction mixture included 0.2 mL enzyme extract and sodium linoleate as the substrate. The homogenates used for enzyme extraction were centrifuged using a GL-20G-II high-speed refrigerated centrifuge (Shanghai Anting Scientific Instrument Factory, Shanghai, China). In addition, the determination of PC-PLC and PI-PLC activities were assayed in accordance with the manufacturer’s protocols for the ELISA kit. The ELISA kits were purchased from Shanghai Enzyme-linked Biotechnology Co., Ltd. (Shanghai, China).

Furthermore, enzymes extraction and activity measurements of SOD, CAT, and APX were conducted following the protocols outlined by Lin et al. [30] and Li et al. [31]. For the determination of SOD activity, its reaction mixture contained 0.1 mL enzyme extract and 3 mL reaction medium, comprising 13 mmol L^−1^ methionine, 0.0015 mmol L^−1^ riboflavin, 0.063 mmol L^−1^ nitro-blue tetrazolium and 50 mmol L^−1^ PBS (pH 7.8). Regarding the assay of CAT activity, its reaction system contained 0.2 mL enzyme extract and 2.8 mL 20 mmol L^−1^ H_2_O_2_. As for APX activity assessment, its reaction mixture included 0.2 mL enzyme extract, 0.5 mL 2 mmol L^−1^ H_2_O_2_ and 4 mL 50 mmol L^−1^ PBS (pH 7.7) supplemented with 0.5 mM ascorbic acid. Moreover, the enzyme extraction and activity assay of POD were performed as per the protocol outlined in Li et al. [31]. Its reaction mixture included 1 mL enzyme extract, 3 mL 50 mmol L^−1^ PBS, 1 mL 50 mmol L^−1^ guaiacol, and 1 mL 2% H_2_O_2_.

The absorbance values for these assays were recorded using the same microplate reader described in Section 2.3, namely an Infinite M200 Pro microplate reader (Tecan, Männedorf, Switzerland). The protein concentration of the above enzyme extract was quantified in accordance with methodology established by Bradford [32]. The activities of both MLMREs and RSEs were presented as U mg^−1^ protein.

### 2.5. Assay of Membrane Lipid Amounts

The protocol of Kuang et al. [10] was utilized to assay the amounts of membrane lipids, including two categories: phospholipids like PC, PA, PI, and DAG, and fatty acids such as USFAs and SFAs. The quantification of phospholipids was carried out following the operational instructions of the corresponding ELISA kit (Shanghai Enzyme-linked Biotechnology Co., Ltd., Shanghai, China). These results were displayed in g kg^−1^. For the measurement of fatty acid amounts, the extract was dissolved in 2 mL 0.5 mol L^−1^ potassium hydroxide (methyl alcohol-dissolved) and 2 mL benzene: petroleum ether (1:1, *v*/*v*). The above solution was shaken, and then 4 mL distilled water was added. The supernatant obtained was employed for the assay of fatty acid levels using an Agilent 7890 gas chromatograph (Agilent Technologies, Inc., Santa Clara, CA, USA) equipped with a flame ionization detector. Separation was performed on a DB-23 capillary column (60 m × 0.32 mm, Agilent Technologies, Inc., Santa Clara, CA, USA). The injection port temperature was maintained at 250 °C. The initial column temperature was set at 50 °C and increased to a final temperature of 245 °C. The detector temperature was set at 280 °C. The flow rates of hydrogen and air were 30 mL min^−1^ and 400 mL min^−1^, respectively. The relative content of each fatty acid was calculated using the peak area normalization method and expressed as the percentage of the individual fatty acid peak area relative to the total peak area of all detected fatty acids. Based on these values, the index of unsaturated fatty acids (IUFA) and the ratio of USFAs to SFAs (U/S) were further calculated.

### 2.6. Assay of ROS-Scavenging Substance Contents

ROS-scavenging substance contents, like AA and GSH, were conducted following Lin et al. [30]. For the assay of AA content, 3 mL of the crude AA extract was mixed with 3 mL 0.5% 1,10-phenanthroline (ethanol-dissolved), 3 mL 5% TCA, 3 mL absolute ethanol, 1.5 mL 0.4% phosphoric acid (ethanol-dissolved), and 1.5 mL 0.03% ferric chloride (ethanol-dissolved). The reaction mixture was incubated at 30 °C for 90 min. In the assay for GSH level, the 1 mL extract was combined with 0.5 mL 4 mmol L^−1^ dithionitrobenzoic acid and 1 mL 50 mmol L^−1^ PBS (pH 7.0). The reaction mixture was incubated at 25 °C for 10 min. The absorbance was measured for AA at 534 nm and for GSH at 412 nm using the same microplate reader. All results were reported as g kg^−1^.

### 2.7. Assay of DPPH Radical-Scavenging Ability

The DPPH radical-scavenging ability was assessed following the protocol reported by Lin et al. [30]. Its reaction mixture consisted of 1.5 mL extract and 1.5 mL 60 μmol L^−1^ DPPH (ethanol-dissolved). The mixture was incubated in the dark at 25 °C for 30 min, and the absorbance was then measured at 517 nm using the same microplate reader. The result was shown as a percentage.

### 2.8. Statistical Analysis

Each data value in the figures is shown as mean ± standard error (*n* = 3). SPSS 21.0 software was used to analyze the acquired data by one-way ANOVA and Duncan’s test. The statistical significance was defined as *p* < 0.01 (represented by **) or *p* < 0.05 (represented by *).

The Pearson correlation analyses between passion fruit’s storage quality and indices associated with membrane lipid modification and ROS-scavenging level were visualized employing the Chiplot Online Tool (https://www.chiplot.online, accessed on 15 March 2026). For the Mantel test [33], the calculations were performed via ggcor (version 0.9.8), and the resulting data were further visualized with the above online tool. For the value of the correlation coefficient (*r*), the ** or * above indicated correlations were significant at *p* < 0.01 or *p* < 0.05, respectively.

## 3. Results

### 3.1. Effects of EBI on Fruit Storage Quality

**Figure 1:**
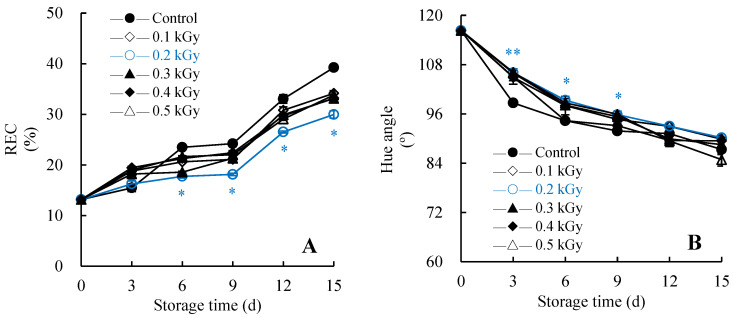

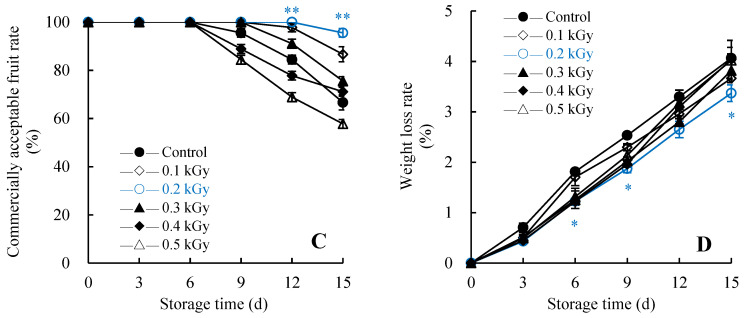
Effects of EBI on the REC (**A**), hue angle (**B**), commercially acceptable fruit rate (**C**) and weight loss rate (**D**) of passion fruit. Value in the figure showed as mean ± standard error (*n* = 3). The asterisks ** (*p* < 0.01) or * (*p* < 0.05) in the figure displayed that there were notable differences between the control group and the 0.2 kGy EBI-treated group on the same storage day. ●, control group; ◇, 0.1 kGy EBI-treated group; ○, 0.2 kGy EBI-treated group; ▲, 0.3 kGy EBI-treated group; ◆, 0.4 kGy EBI-treated group; △, 0.5 kGy EBI-treated group.

Quick increases in REC (Figure 1A) and weight loss rate (Figure 1D) were shown in the control fruit. However, EBI-treated passion fruit maintained lower levels of REC and weight loss rate, particularly in EBI group with a dose of 0.2 kGy. Moreover, substantial differences in REC between the control fruit and 0.2 kGy EBI-treated fruit were observed on 6–15 d, whereas notable differences in weight loss rate between these two groups were displayed on 6–9 d and 15 d.

The hue angle of passion fruit showed a consistent decline during storage (Figure 1B). By contrast, passion fruit treated with 0.2 kGy EBI kept a higher level than the other groups. Besides, between 3 d and 9 d, 0.2 kGy EBI-treated fruit had a clearly higher hue angle compared to the control fruit.

As shown in Figure 1C, the commercially acceptable fruit rate of the control and the 0.4 and 0.5 kGy EBI-treated fruit began to decrease from day 6, whereas that of the 0.1 and 0.3 kGy EBI-treated fruit decreased from day 9 to day 15. However, the 0.2 kGy EBI-treated group exhibited no decline until 12 d, with this decreasing trend persisting through to 15 d. Moreover, between 12 d and 15 d, the 0.2 kGy EBI-treated fruit kept a clearly higher level compared to the control samples.

### 3.2. Effects of EBI on O_2_^−.^ Production Rate and MDA Content

Given that EBI at a dose of 0.2 kGy was identified as the most effective for preserving the storage quality of passion fruit, subsequent research centered on unraveling the mechanisms by which this specific EBI treatment improved the passion fruit storage quality by regulating membrane lipid composition and ROS-scavenging capacity.

**Figure 2:**
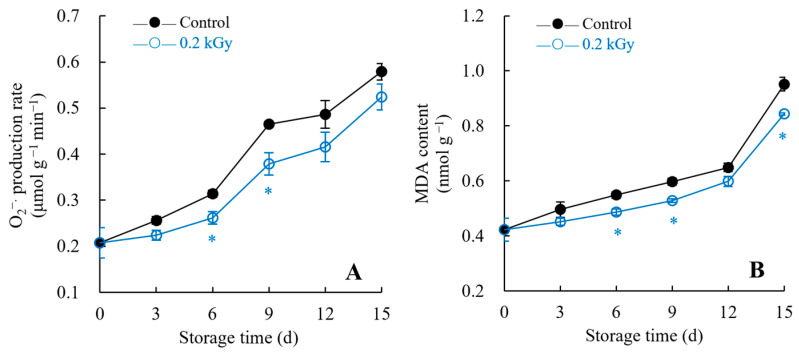
Effects of EBI on the production rate of O_2_^−.^ (**A**) and content of MDA (**B**) of passion fruit. Value in the figure showed as mean ± standard error (*n* = 3). The asterisk * (*p* < 0.05) in the figure displayed that there was a notable difference between the control group and the EBI-treated group on the same storage day. ●, control group; ○, EBI-treated group.

As shown in Figure 2A, the O_2_^−.^ production rate of passion fruit increased gradually on 0–15 d, with the level in the EBI-treated group remaining lower than that in the control group. Meanwhile, significant differences in the O_2_^−.^ production rate between the two groups were shown from 6 d to 9 d. Compared with the control fruit, EBI treatment resulted in a 10.34% decrease of O_2_^−.^ production rate at 15 d.

Figure 2B revealed that the MDA value of the control samples was in the range of 0.42–0.95 nmol g^−1^. After EBI treatment, the MDA value of fruit was in the range of 0.42–0.84 nmol g^−1^. Besides, the EBI-treated samples held a remarkably lower MDA amount than the control samples on 6–9 d and 15 d.

### 3.3. Effects of EBI on MLMRE Activities

**Figure 3:**
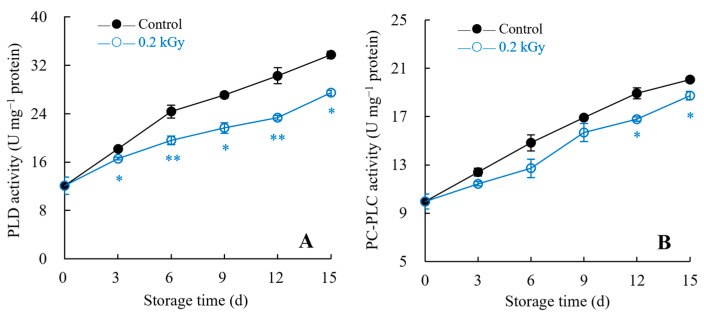

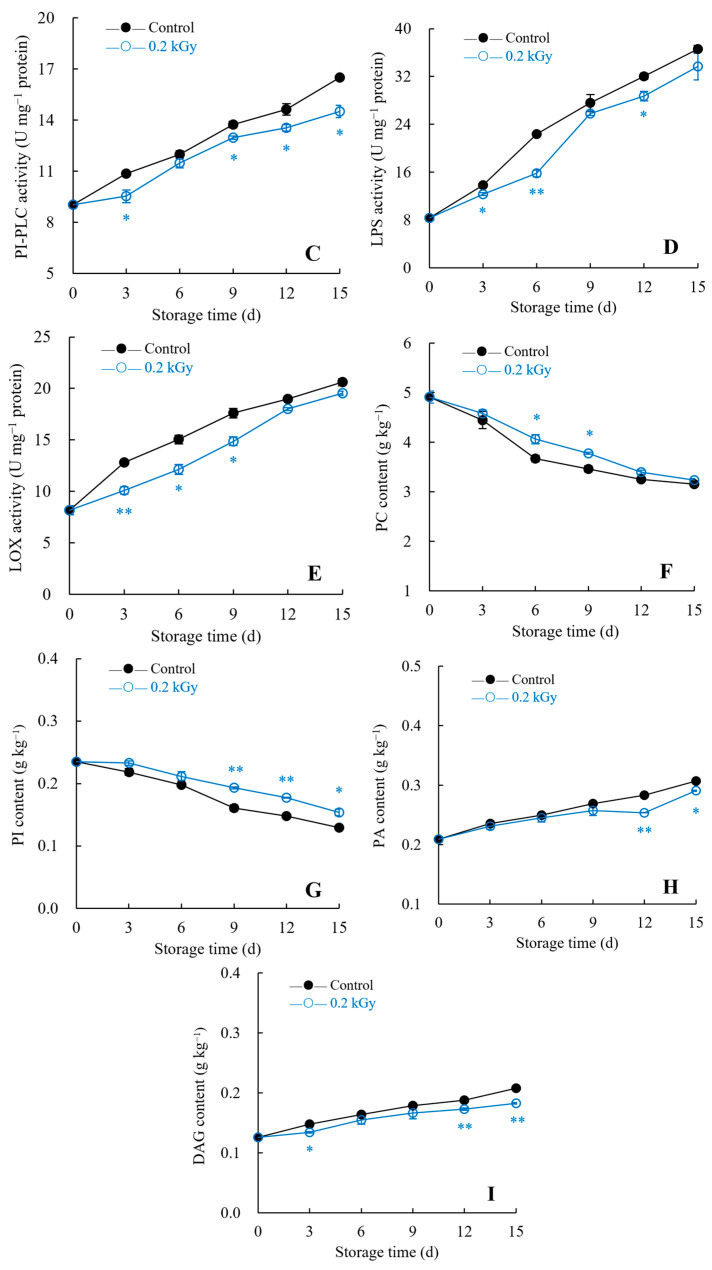
Effects of EBI on the activities of PLD (**A**), PC-PLC (**B**), PI-PLC (**C**), LPS (**D**) and LOX (**E**), and contents of PC (**F**), PI (**G**), PA (**H**), and DAG (**I**) of passion fruit. Value in the figure showed as mean ± standard error (*n* = 3). The asterisks ** (*p* < 0.01) or * (*p* < 0.05) in the figure displayed that there were notable differences between the control group and the EBI-treated group on the same storage day. ●, control group; ○, EBI-treated group.

The MLMRE activities of passion fruit, containing PLD (Figure 3A), PC-PLC (Figure 3B), PI-PLC (Figure 3C), LPS (Figure 3D), and LOX (Figure 3E), exhibited an upward trend. On the other hand, the rate of increase of these enzyme activities was lower in the EBI-treated passion fruit during 0–15 d. Moreover, EBI-treated samples revealed prominently lower activities of PLD on 3–15 d, PC-PLC on 12–15 d, PI-PLC on 3 d and 9–15 d, LPS on 3–6 d and 12 d, and LOX on 3–9 d, respectively. Furthermore, compared to the control passion fruit, the activities of these enzymes in the EBI-treated group on 15 d were 18.59%, 6.53%, 12.17%, 8.08%, and 5.14% lower, respectively.

### 3.4. Effects of EBI on Phospholipid Contents

The PC (Figure 3F) and PI (Figure 3G) contents in passion fruit decreased rapidly during 0–15 d. For PC content, the control fruit and EBI-treated fruit descended from 4.91 g kg^−1^ (0 d) to 3.16 and 3.23 g kg^−1^ (15 d), respectively. Moreover, the EBI-treated group retained a markedly greater value than the control group at 6–9 d. For PI content, these two groups dropped from 0.24 g kg^−1^ (0 d) to 0.13 and 0.15 g kg^−1^ (15 d), respectively. Furthermore, the EBI-treated group maintained a considerably greater value than the control group on 9–15 d.

The PA (Figure 3H) and DAG (Figure 3I) levels in passion fruit samples rose quickly within 0–15 d. For PA content, the control fruit and EBI-treated fruit rose from 0.21 g kg^−1^ (0 d) to 0.31 and 0.29 g kg^−1^ (15 d), respectively. Furthermore, EBI-treated fruit held a significantly lower value than control samples on 12–15 d. For DAG content, the control group and EBI-treated group increased from 0.13 g kg^−1^ (0 d) to 0.21 and 0.18 g kg^−1^ (15 d), respectively. Besides, EBI-treated fruit maintained a clearly lower value than the control samples on 3 d and 12–15 d.

### 3.5. Effects of EBI on Fatty Acid Contents

**Figure 4:**
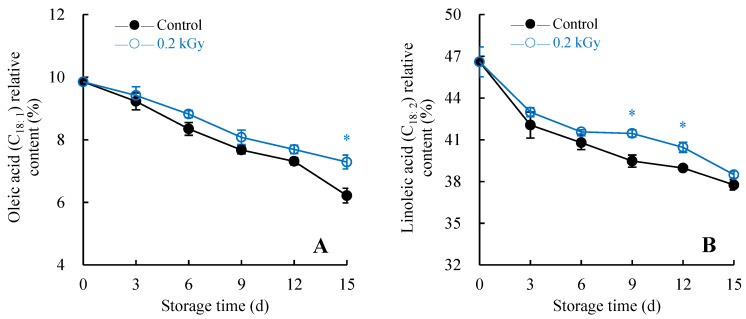

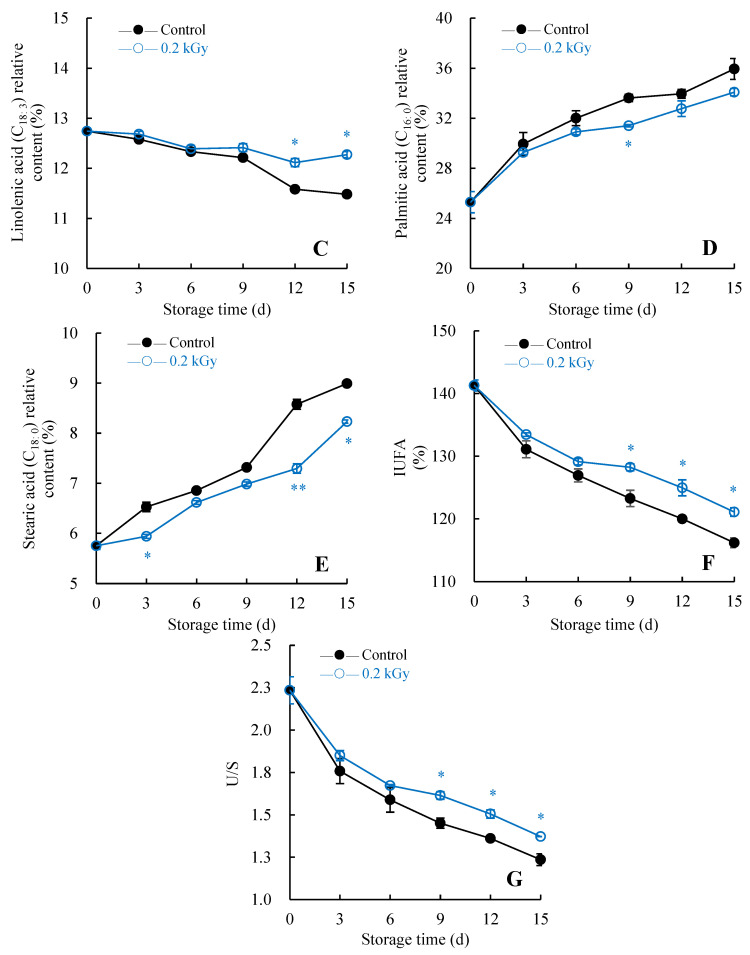
Effects of EBI on the relative contents of USFAs (**A**–**C**) and SFAs (**D**,**E**), and levels of IUFA (**F**) and U/S (**G**) of passion fruit. Value in the figure showed as mean ± standard error (*n* = 3). The asterisks ** (*p* < 0.01) or * (*p* < 0.05) in the figure displayed that there were notable differences between the control group and the EBI-treated group on the same storage day. ●, control group; ○, EBI-treated group.

The relative amounts of USFAs, including oleic acid (Figure 4A), linoleic acid (Figure 4B), and linolenic acid (Figure 4C), of passion fruit exhibited a reducing trend during storage, whereas EBI-treated fruit showed higher levels than the control fruit throughout storage. Specifically, in contrast to the control passion fruit, EBI-treated fruit retained markedly higher relative contents of oleic acid on 15 d, linoleic acid on 9–12 d, and linolenic acid on 12–15 d, respectively. For example, on 15 d, the relative contents of these USFAs in the EBI-treated group were 1.17, 1.02, and 1.07 times those in the control samples, respectively.

The relative levels of SFAs, like palmitic acid (Figure 4D) and stearic acid (Figure 4E), of passion fruit followed an upward trend from 0 d to 15 d. However, EBI-treated samples retained lower values than control samples. Specifically, compared with the control group, the EBI-treated group exhibited clearly lower relative contents of palmitic acid on 9 d as well as stearic acid on 3 d and from 12 d to 15 d, respectively. For instance, on 15 d, the relative contents of these SFAs in the EBI-treated group were 94.83% and 91.55% of those in control fruit, respectively.

### 3.6. Effects of EBI on IUFA and U/S

The IUFA of the control samples and EBI-treated samples ranged from 141.29% to 116.18% and 121.09%, respectively (Figure 4F). By contrast, the U/S of the control fruit and EBI-treated fruit ranged from 2.23 to 1.24 and 1.37, respectively (Figure 4G). Additionally, remarkable changes in IUFA and U/S were revealed between the EBI-treated fruit and control fruit from 9 d to 15 d.

### 3.7. Effects of EBI on ROS-Scavenging Enzyme Activities

**Figure 5:**
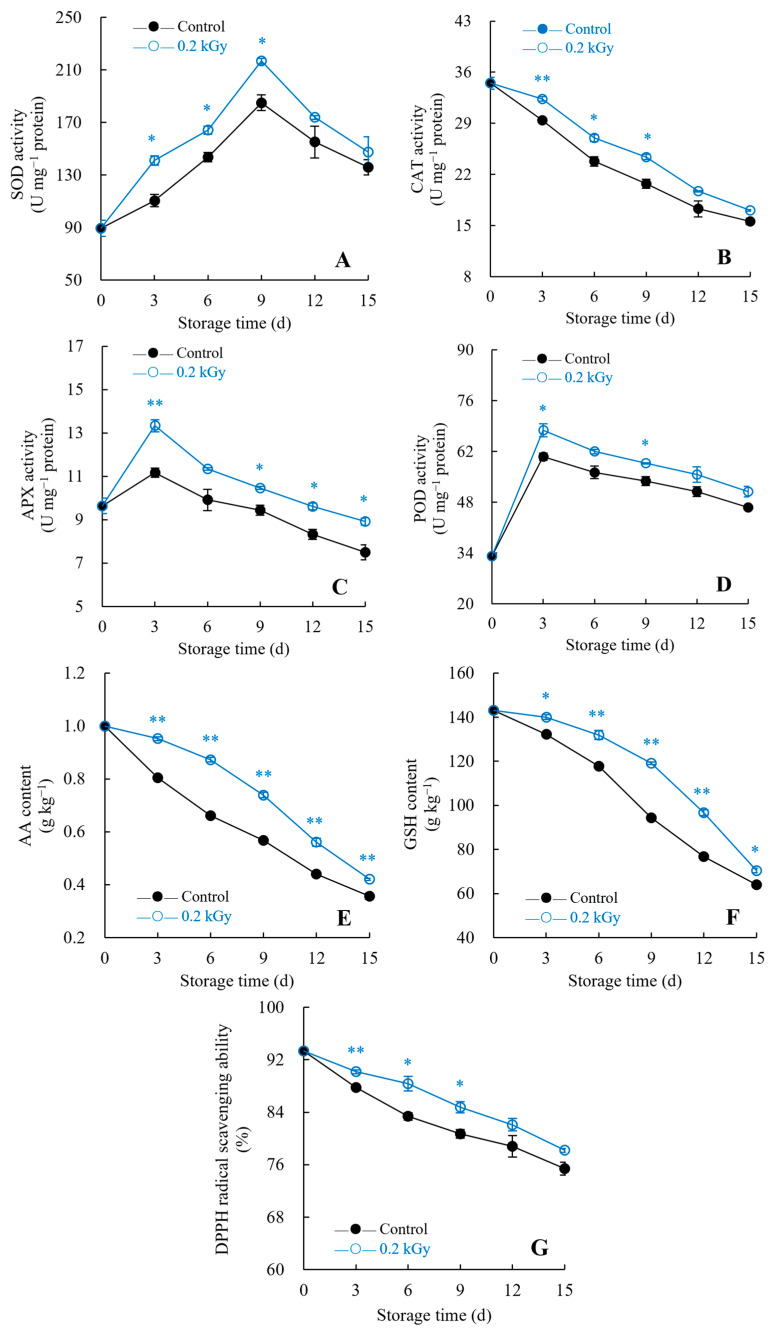
Effects of EBI on the activities of SOD (**A**), CAT (**B**), APX (**C**), and POD (**D**), contents of AA (**E**) and GSH (**F**), and scavenging ability of DPPH radical (**G**) of passion fruit. Value in the figure showed as mean ± standard error (*n* = 3). The asterisks ** (*p* < 0.01) or * (*p* < 0.05) in the figure displayed that there were notable differences between the control group and the EBI-treated group on the same storage day. ●, control group; ○, EBI-treated group.

The SOD activity in both groups revealed a trend of first rising and then declining (Figure 5A), with peak activity appearing on 9 d. By contrast, the EBI-treated group remained at a higher level, with its peak being 1.17 times that of the control group. Moreover, significant differences between the two groups were observed on 3–9 d.

The results are displayed in Figure 5B, showing that the CAT activity of passion fruit reduced gradually until 15 d. On 15 d, compared with 0 d, the CAT activity in the control group and the EBI-treated group had decreased by 6.42% and 5.08%, respectively. Moreover, clear differences between the two groups were revealed on 3–9 d.

Figure 5C exhibits that the APX activity in the two groups followed a similar trend: rising during 0–3 d, and then decreasing within 3–15 d. The key difference between them was that the peak APX activity of the control group was 11.17 U mg^−1^ protein, while that of the EBI-treated group was 13.33 U mg^−1^ protein. Furthermore, the EBI-treated group showed notably higher activity than the control group at 3 d and 9–15 d.

Figure 5D displays that POD activity in the control fruit raised at 0–3 d, peaking at 60.50 U mg^−1^ protein, before reducing from 3 d. However, EBI-treated fruit maintained a higher level of activity than the control fruit, with clear differences observed at 3 d and 9 d.

### 3.8. Effects of EBI on ROS-Scavenging Substance Contents

Both groups exhibited a rapid decline in the contents of AA (Figure 5E) and GSH (Figure 5F). However, the EBI-treated fruit maintained higher levels of these substances than the control fruit, with obvious differences observed from 3 d to 15 d. In particular, on 15 d, the amounts of these two substances in the EBI-treated group were 1.17 and 1.10 times those in the control group, respectively.

### 3.9. Effect of EBI on DPPH Radical-Scavenging Ability

Figure 5G reveals that DPPH radical-scavenging ability in the control samples and EBI-treated samples ranged from 93.32% to 75.41% and 78.19%, respectively. Significant changes were observed for the EBI-treated group versus the control group during 3–9 d. In addition, the initial values of DPPH radical-scavenging ability in the control group and EBI-treated group were 1.24 and 1.19 times those at 15 d, respectively.

### 3.10. Correlation Analyses

**Figure 6:**
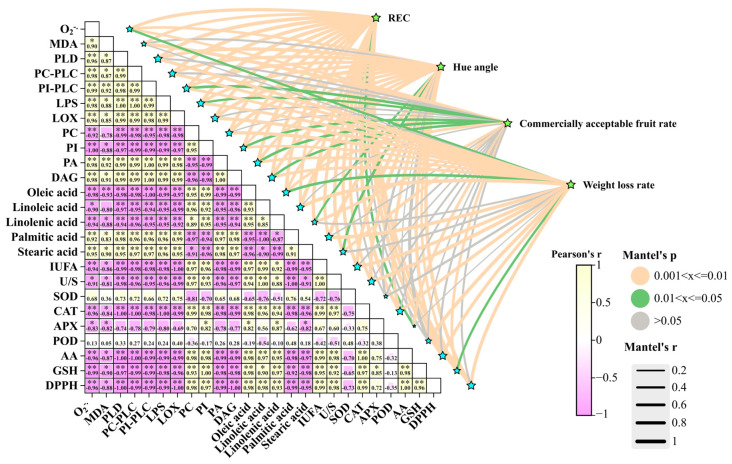
Mantel test for correlations between storage quality and indices related to membrane lipid compositions and ROS-scavenging potential in passion fruit. The five-pointed stars represent variables in the Mantel correlation network. Green stars indicate storage quality attributes, while blue stars indicate physiological and biochemical indices; each blue star corresponds to the index in the same row of the Pearson correlation matrix. Star size has no specific statistical meaning unless otherwise stated. Curved links represent Mantel correlations, with line color and width indicating Mantel’s p and r values, respectively. In the Pearson correlation matrix, * and ** indicate significance at *p* < 0.05 and *p* < 0.01, respectively; the color indicate the statistical significance, and color intensity shows the correlation strength (purple: negative, yellow: positive).

To examine the relationships between the storage quality and parameters associated with changes in membrane lipid levels and ROS-scavenging potential of passion fruit, the Mantel test analysis was implemented. As presented in Figure 6, REC exhibited a strong correlation with all other indicators except for SOD and POD activities. Similarly, the hue angle was strongly correlated with all other indicators, excluding MDA content, and SOD, APX, and POD activities. Weight loss rate was strongly correlated with all other indicators, apart from levels of MDA, linolenic acid, stearic acid, SOD, and APX. Additionally, commercially acceptable fruit rate was strongly correlated with the contents of O_2_^−.^, MDA, PI, PA, DAG, oleic acid, linolenic acid, stearic acid, and GSH, as well as the activities of PI-PLC, LPS, and APX. Therefore, the Mantel test suggested that the changed membrane lipid levels and ROS-scavenging potential could be two crucial regulators of storage quality in passion fruit.

Furthermore, Figure 6 also displays that the PLD level was negatively correlated with the value of PC (*r* = −0.99), while positively correlated with levels of PA (*r* = 0.99) and DAG (*r* = 0.99), respectively. DAG content was positively correlated with levels of PC-PLC (*r* = 0.99) and PI-PLC (*r* = 1.00). PC or PI contents were negatively correlated with values of PC-PLC (*r* = −0.98) or PI-PLC (*r* = −0.99). LOX activity showed negative correlations with levels of oleic acid (*r* = −0.97), linoleic acid (*r* = −0.99), linolenic acid (*r* = −0.92), IUFA (*r* = −1.00), and U/S (*r* = −0.99), but revealed a positive correlation with the content of MDA (*r* = 0.85), respectively. Moreover, MDA content showed a negative correlation with levels of CAT (*r* = −0.84), APX (*r* = −0.82), AA (*r* = −0.87), GSH (*r* = −0.90), and DPPH radical-scavenging ability (*r* = −0.88), respectively. Furthermore, O_2_^−.^ production rate was positively correlated with the contents of PA (*r* = 0.98) and MDA (*r* = 0.90) but negatively correlated with the values of AA (*r* = −0.96), GSH (*r* = −0.99) and DPPH radical-scavenging ability (*r* = −0.96), respectively. The findings indicated that higher MLMRE activities were identified as potential factors underlying variations in membrane lipid contents. Meanwhile, a decline in ROS-scavenging substance contents (such as GSH and AA) and reduced DPPH radical-scavenging ability in passion fruit might result in ROS buildup and membrane lipid peroxidation. These factors collectively destroyed the cell membranes and negatively impacted the storage quality of passion fruit.

## 4. Discussion

Passion fruit is prone to quality deterioration under prolonged exposure to high temperature [34]. Water loss, wrinkling, and pathogen infection are major factors contributing to fruit spoilage and quality loss in harvested passion fruit [1,7]. Previous studies have shown that EBI can reduce postharvest losses and delay the fruit ripening and senescence [26,35]. In this study, among the tested EBI doses, 0.2 kGy EBI most effectively preserved the key quality traits of passion fruit, as evidenced by the lower REC and weight loss rate, while showing a higher hue angle and commercially acceptable fruit rate during storage. On day 15 of storage, the REC, hue angle, commercially acceptable fruit rate, and weight loss rate of the control group were 39.24%, 87.34°, 66.67%, and 4.07%, respectively. In contrast, 0.2 kGy EBI-treated passion fruit had corresponding values of 29.95%, 90.21°, 95.56%, and 3.37%, respectively. These data indicate that 0.2 kGy EBI reduced REC and weight loss rate by 23.67% and 17.20%, respectively, while increasing the hue angle and commercially acceptable fruit rate by 3.29% and 43.33%, respectively, compared with the control. Therefore, the preservation effect of 0.2 kGy EBI was not only statistically significant but also quantitatively evident across multiple quality-related indicators. Accordingly, 0.2 kGy was chosen for subsequent investigations to determine whether the EBI-mediated improvement in passion fruit storage quality was associated with changes in membrane lipid composition and ROS-scavenging capacity. Consistent with our results, previous studies had reported similar influences of postharvest treatment of EBI on mango fruit [26] and lime fruit [25].

During postharvest storage, ROS accumulation in fruit may be derived from multiple metabolic pathways, including enhanced respiration, mitochondrial electron leakage, membrane-associated oxidase activity, lipid oxidation, and senescence-related disruption of cellular redox homeostasis [5,21]. ROS-mediated attack on cell membranes promotes lipid peroxidation, resulting in MDA accumulation and subsequent aggravation of membrane damage [5]. This research revealed that EBI suppressed the raised O_2_^−.^ production rate, reduced MDA accumulation, and delayed the increase in REC in passion fruit. Furthermore, compared to untreated passion fruit, the fruit treated with EBI maintained a higher hue angle and commercially acceptable fruit rate but a lower weight loss rate. Consequently, EBI decreased the ROS accumulation, and alleviated lipid peroxidation, and thus retained the cell membrane structures and contributed to the improvement of the storage quality of passion fruit. Accumulating evidence also indicated that the reduced ROS accumulation and minor lipid peroxidation in fruits, including longans [12], peaches [22], and winter jujubes [9], were associated with cellular membrane stability and improved postharvest quality.

Excessive ROS may induce damage to the cell membranes. The cell membrane serves as the primary defensive barrier against external biotic and abiotic stress [9]. The cell membrane structure is changed by the synergistic catalytic effects of MLMREs like PLD, PC-PLC, LPS, PI-PLC, and LOX, as reflected by modifications in membrane lipids such as phospholipids and fatty acids [13,14]. Phospholipids serve as the main structural components of cell membrane and key participants of cellular information transmission [9]. As the two primary phospholipids, PC and PI play pivotal roles in preserving cell membrane stability [10]. The hydrolytic degradation of PC is mediated by PLD, producing DAG and PA in this process [15,36]. Excessive PA will improve the ROS production by activating nicotinamide adenine dinucleotide phosphate (NADPH) oxidase, thereby impairing cell membrane structure [9,36]. PLC may be divided into PC-PLC or PI-PLC. PC-PLC can hydrolyze PC, leading to the formation of DAG, whereas PI undergoes cleavage through PI-PLC, producing DAG [13,15]. This study exhibited that, compared to control fruit, EBI-treated fruit maintained lower levels of PLD, PC-PLC, and PI-PLC within storage, accompanied by higher amounts of PC and PI, as well as lower contents of PA, DAG, and O_2_^−.^ Furthermore, the storage quality of EBI-treated passion group was superior to that of the control group at 0–15 d. These experimental results hinted that EBI inhibited the degradation of primary phospholipids (PC, PI) by reducing PLD, PC-PLC, and PI-PLC activities. This not only suppressed the accumulation of degradation products (PA, DAG) and ROS-mediated oxidative damage, but also retained the cell membrane integrity, thereby preserving storage quality in passion fruit. Consistent with the observation of Yang et al. [9], their results revealed that either methyl jasmonate or methyl salicylate could suppress PLC and PLD activities, which mitigate the decreases in PC and PI levels but preserve the storage quality of winter jujube. Similarly, Zhang et al. [16] revealed that spermidine sustained elevated contents of PC and PI by lowering PLC and PLD activities, thus preserving the cell membranes and delaying the senescence in prune fruit.

In addition, SFAs and USFAs constitute the two major categories of fatty acids [5]. LPS is capable of hydrolyzing DAG and PA to catalyze the generation of FFAs like USFAs or SFAs [13], as well as releasing the fatty acids from the membrane lipids [16]. Compared with control passion fruit, EBI inhibited the LPS activity of passion fruit, which attenuated the release of fatty acids and contributed to the preservation of cell membrane stability. Furthermore, changes in contents of USFAs and SFAs influence the integrity and fluidity of cell membranes, with such effects being linked to fatty acid chain length and unsaturation degree [16]. Increased USFA contents in cell membranes can facilitate the material transport and signal transduction [16]. Notably, LOX can modulate the catalysis of USFAs into SFAs, accompanied by MDA production and membrane damages. These effects ultimately lead to changes in fatty acid unsaturation, where IUFA and U/S act as reliable evaluation indicators [5]. In this research, EBI-treated fruit had lower LOX activity than non-EBI-treated fruit during storage. Additionally, the treated fruit presented greater relative levels of oleic acid, linoleic acid, and linolenic acid, higher IUFA and U/S, but lower relative levels of palmitic acid and stearic acid, and a lower MDA content. These results demonstrated that EBI-mediated inhibition of LOX activity not only increased USFA accumulation, promoted SFA desaturation, and enhanced fatty acid unsaturation, but also suppressed lipid peroxidation. These effects collectively contributed to the maintenance of membrane integrity and preservation of storage quality in passion fruit, which were consistent with a higher hue angle and commercially acceptable fruit rate, but a lower REC and weight loss rate. These results were aligned with the research of Xie et al. [36], which displayed that lower LOX activity might increase the USFA contents and maintain the cell membrane integrity, ultimately alleviating chilling injury and retaining the quality of muskmelons. Furthermore, Ding et al. [37] reported that phytosulfokine-α helped preserve membrane integrity through the suppression of LOX activity and increase in the proportion of USFAs, thereby leading to delayed senescence and improved quality of *Rosa roxburghii* fruit. Therefore, regulation of membrane lipid composition may be one of the key mechanisms underlying the EBI-mediated improvement in passion fruit storage quality.

Furthermore, a lower ROS level is conducive to stabilizing the cell membrane structure [5]. During evolution, plants have developed specialized defense systems for scavenging ROS, consisting of the core enzymatic (APX, SOD, POD, and CAT) and non-enzymatic (AA and GSH) systems [1,22,38]. Excessive O_2_^−.^ is dismutated by SOD to form H_2_O_2_, which is then converted into H_2_O by cooperative actions of POD and CAT, thereby helping preserve the ROS homeostasis and attenuating damages to cellular membranes [16,17]. Furthermore, APX as well as non-enzymatic compounds (e.g., AA and GSH) involved in the AA-GSH cycle exert a pivotal function in ROS elimination [16]. The effect of EBI on ROS metabolism may be associated with a low-dose stress-response mechanism. Electron beam irradiation can generate transient oxidative signals through ionization and water radiolysis; however, an appropriate dose may activate endogenous defense responses rather than cause oxidative injury. The 0.2 kGy EBI treatment appeared to induce such a protective response, as reflected by enhanced activities of SOD, CAT, APX, and POD. SOD represents the first enzymatic defense line by converting O_2_^−.^ into H_2_O_2_, while CAT, APX, and POD further detoxify H_2_O_2_ into H_2_O, thereby reducing the possibility of secondary radical formation. In addition to enzymatic antioxidants, AA can serve as an electron donor for APX-mediated H_2_O_2_ removal, whereas GSH participates in redox cycling and ROS detoxification [16]. The higher AA and GSH contents observed in EBI-treated fruit suggest that EBI helped maintain the non-enzymatic antioxidant pool. Accordingly, the higher DPPH radical-scavenging ability in EBI-treated fruit reflected an improved overall hydrogen- or electron-donating capacity of fruit extracts. Although DPPH does not directly represent specific ROS species in vivo, it provides useful complementary evidence for the enhanced antioxidant potential of EBI-treated passion fruit. Taken together, these results suggest that EBI improved ROS-scavenging capacity through coordinated regulation of enzymatic antioxidants, non-enzymatic antioxidants, and radical-quenching potential, thereby reducing ROS accumulation, limiting membrane lipid peroxidation, and maintaining storage quality. Consistent with our findings, studies on EBI-treated winter jujube fruit [24], trisodium phosphate-treated passion fruit [6], and carvacrol-treated peach fruit [22] have validated that higher levels of RSEs or antioxidants were linked to augmented ROS-scavenging potential and superior fruit quality. Consequently, regulating ROS-scavenging potential of passion fruit represented another critical mechanism underlying the improvement in fruit storage quality via EBI.

## 5. Conclusions

**Figure 7:**
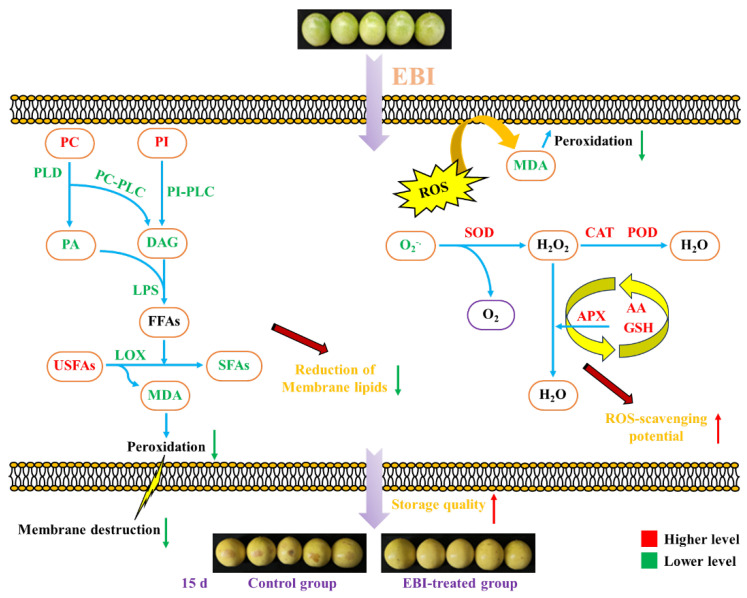
The possible mechanism of EBI improving the storage quality of passion fruit by regulating membrane lipid compositions and enhancing ROS-scavenging potential. In the figure, compared with the control group, the green fonts show that the EBI-treated group had lower levels of indicators, but red fonts show that the EBI-treated group had higher levels of indicators.

The preservation effect of 0.2 kGy EBI was not limited to improvements in external quality attributes, including reduced REC and weight loss rate and increased hue angle and commercially acceptable fruit rate. More importantly, these quality improvements were closely associated with the maintenance of membrane lipid stability and the enhancement of ROS-scavenging capacity. A proposed model is depicted in Figure 7. Treatment with 0.2 kGy EBI lowered the activities of MLMREs (LOX, PI-PLC, LPS, PLD, PC-PLC), slowed down the reductions in the contents of USFAs and phospholipids (PC, PI), and diminished the production of PA, DAG, SFAs, and lipid peroxidation metabolites. These effects contributed to preserving the degree of fatty acid unsaturation and the structural integrity of cellular membranes in passion fruit. Furthermore, 0.2 kGy EBI sustained higher levels of antioxidants (AA, GSH), RSEs (SOD, POD, APX, CAT) and DPPH radical-scavenging capacity, while suppressing the accumulation of ROS and MDA. These responses may alleviate membrane damage in passion fruit during storage. Therefore, the mechanistic findings provide a physiological basis for explaining the practical preservative effect of EBI treatment. Nevertheless, future studies incorporating sensory evaluation, microbial population analysis, shelf-life prediction models, and economic feasibility assessment would further support the practical application of EBI technology in passion fruit preservation. Further investigations into additional ROS-related indicators, microscopic characteristics, and the expression profiles of key genes involved in membrane lipid metabolism and antioxidant defense would help clarify the molecular regulatory mechanisms underlying EBI-mediated preservation of passion fruit.

## Data Availability

The original contributions presented in this study are included in the article. Further inquiries can be directed to the corresponding authors.

## Abbreviations

The following abbreviations are used in this manuscript:

AA	ascorbic acid
ANOVA	analysis of variance
APX	ascorbate peroxidase
CAT	catalase
DAG	diacylglycerol
DPPH	1,1-diphenyl-2-picrylhydrazyl
EBI	electron beam irradiation
FFAs	free fatty acids
GSH	glutathione
H_2_O_2_	hydrogen peroxide
IUFA	index of unsaturated fatty acids
LOX	lipoxygenase
LPS	lipase
MDA	malondialdehyde
MLMREs	membrane lipid metabolism-related enzymes
NADPH	nicotinamide adenine dinucleotide phosphate
O_2_^−.^	superoxide anion
PA	phosphatidic acid
PBS	phosphate buffered saline
PC	phosphatidylcholine
PC-PLC	PC-specific PLC
PLA	phospholipase A
PLC	phospholipase C
PLD	phospholipase D
PI	phosphatidylinositol
PI-PLC	PI-specific PLC
POD	peroxidase
REC	relative electrical conductivity
ROS	reactive oxygen species
RSEs	ROS-scavenging enzymes
SFAs	saturated fatty acids
SOD	superoxide dismutase
TCA	trichloroacetic acid
USFAs	unsaturated fatty acids
U/S	ratio of USFAs to SFAs
